# Unmasking of pre-excitation after aortic valve surgery – A report of two cases

**DOI:** 10.1016/j.hrcr.2020.12.006

**Published:** 2020-12-21

**Authors:** Jari M. Tapanainen, Per Insulander, Nikola Drca, Anette Jemtrén, Finn Åkerström, Mats Jensen-Urstad

**Affiliations:** Department of Cardiology, Karolinska Institute at Karolinska University Hospital, Stockholm, Sweden

**Keywords:** Aortic valve surgery, AV conduction, AV node, Pre-excitation, WPW

## Introduction

Wolff-Parkinson-White syndrome is defined as a combination of pre-excitation in the electrocardiogram (ECG) and symptoms related to tachyarrhythmia. The pre-excitation can be intermittent, and thus is not always present in ECG. The presence and degree of pre-excitation is dependent on characteristics of conduction through the atrioventricular (AV) node, which in turn can be affected by autonomous tone, medication, a conduction system disease, or mechanical injury. In addition to adenosine provocation test, unmasking of an accessory pathway (AP) has been reported during anesthesia,^1^^,^^2^ in conjunction with His bundle ablation,^3^ and after tricuspid valve replacement.^4^ There is a recent case report on a transient unmasking of an AP after aortic valve replacement.^5^ Some APs present with malignant properties, ie, they may predispose to ventricular fibrillation and sudden cardiac death because of the very short refractory period in the AP permitting remarkably fast antegrade conduction during a potential episode of atrial fibrillation.^6^

We report 2 cases involving patients in whom previously unrecognized pre-excitation was unmasked after aortic valve surgery. The incidence of newly discovered pre-excitation after aortic valve surgery or transcatheter aortic valve replacement is not known.

## Case report

### Case 1

**Figure 1:**
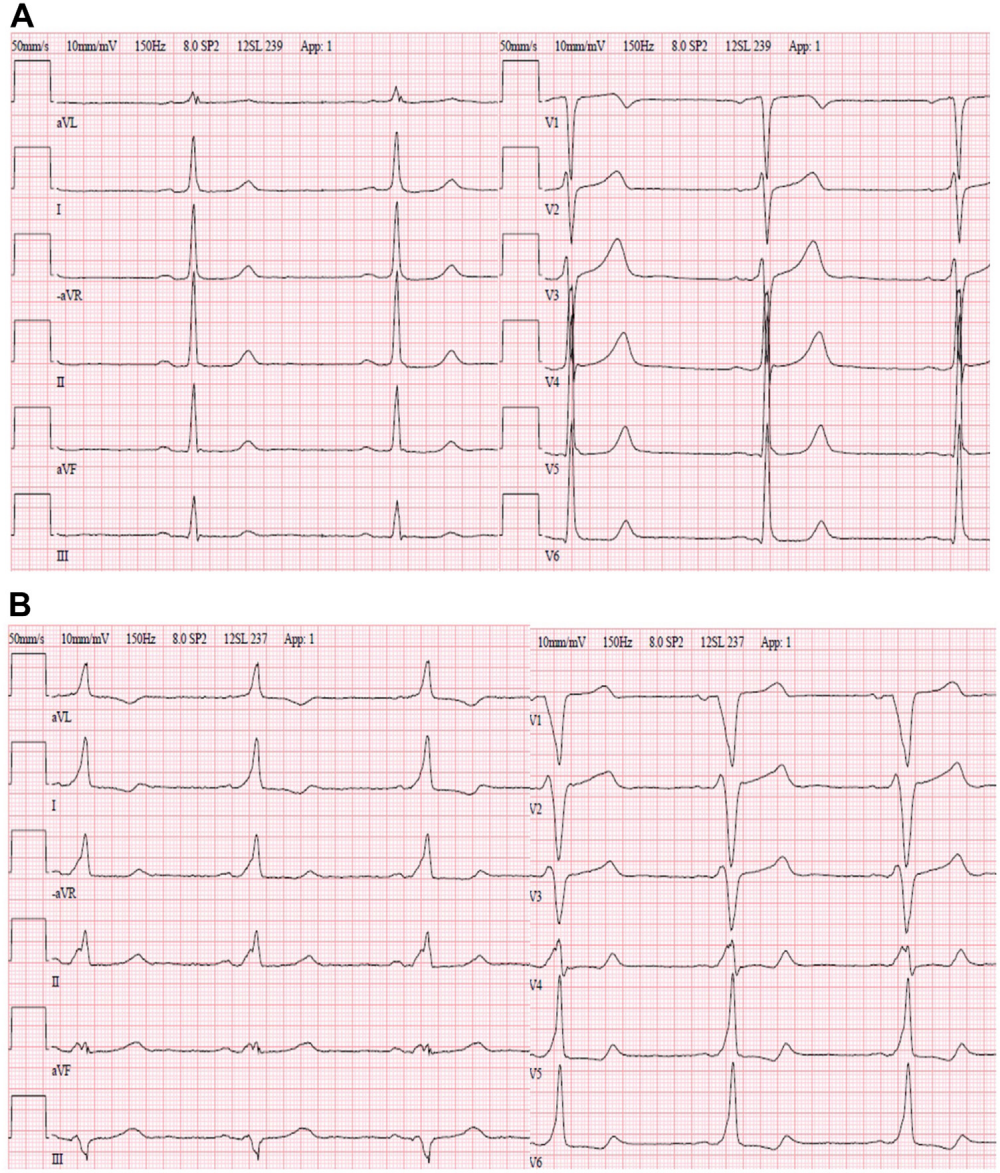
Case 1. **A:** Electrocardiogram (ECG) prior to aortic valve surgery with no clear pre-excitation. **B:** ECG after 15 days after aortic valve replacement.

A 30-year-old man was referred for echocardiography because of a systo-diastolic murmur. He was diagnosed with a significant aortic valve stenosis (maximum velocity 3 m/s) and regurgitation, and was referred for cardiac surgery. Prior to surgery, his ECG was normal with no clear pre-excitation (Figure 1A). The aortic valve was bicuspid and calcified during surgery. The aortic root was dilated at the sinus of Valsalva (47 mm) and the ascending aorta was dilated with a maximum diameter of 40 mm. The valve was replaced with a 23 mm mechanical prosthetic valve and a composite graft in the ascending aorta. No perioperative complications occurred. Postoperative medication included metoprolol 50 mg per day. The ECG at discharge showed intermittently a normofrequent nodal rhythm with retrograde P waves or sinus rhythm with a first-degree AV block with an extremely long PQ interval. The patient was readmitted to hospital 15 days after the operation because of dizziness and chest pain. His ECG now revealed sinus rhythm and pre-excitation suggesting a right-sided midseptal AP (Figure 1B). Echocardiography showed a mechanical aortic valve prosthesis with a normal function, septal hypokinesia, and a pericardial effusion without hemodynamic effect. Metoprolol was discontinued. Exercise ECG disclosed a working capacity of 212 W during sinus rhythm with a pulse response from 62 to 173 beats/min and consistent pre-excitation. The patient was referred for an electrophysiological (EP) study, which was performed 6 months after the surgery.

During the EP study, catheters were placed in the coronary sinus, along the His bundle, and in the right ventricular apex. Partial pre-excitation was demonstrated at baseline with PQ 124 ms, AH 74 ms, and HV 30 ms. Ventricular pacing at 700 ms cycle length lead to ventriculoatrial (VA) dissociation. The pre-excitation disappeared during atrial pacing from the coronary sinus. Incremental atrial pacing revealed frequency-dependent left-sided bundle branch block at the pacing cycle length of 580 ms and a Wenckebach point of 310 ms. During isoproterenol challenge no pre-excitation was seen, and incremental ventricular pacing disclosed decremental retrograde AV nodal conduction with a VA block at 610 ms. The AP was not ablated in the absence of malignant features or symptoms that could have been attributed to the pathway.

### Case 2

**Figure 2:**
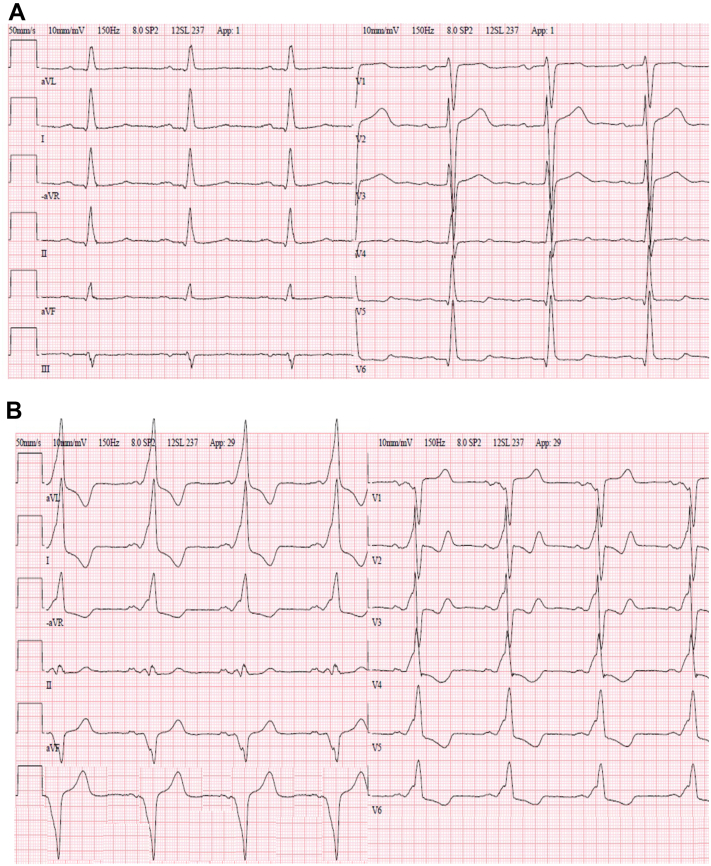
Case 2. **A:** Electrocardiogram (ECG) prior to aortic valve surgery with no pre-excitation. **B:** ECG 9 years after the first operation (3 years after the second operation and after repeated cardioversions because of atrial fibrillation).

A 38-year-old man with a history of hypertension and a known combined aortic valve vitium since the age of 12 was referred for aortic valve replacement surgery because of reduced physical capacity, exercise-induced dyspnea, and syncope. On echocardiography, the aortic valve was calcified with a valve area of 1 cm^2^ and a maximal gradient of 105 mm Hg. Aortic regurgitation was graded to be moderately severe (grade III/IV). The left ventricular systolic function was reduced, with an ejection fraction of 35%. His ECG showed normal sinus rhythm with no pre-excitation (Figure 2A). Perioperatively, the aortic valve proved to be bicuspid and severely calcified. The valve was excided and the annulus was decalcified, and a 27 mm Medtronic Hall mechanical prosthetic valve was used to replace the defective valve. No bradycardia was documented after the surgery. Six years later, the patient was reoperated with a 32 mm Hemashield graft because of an aneurysm in the ascending aorta. After the second surgery he developed problems with recurring paroxysmal and persistent atrial fibrillation and he had to undergo repeated DC cardioversions. Three years later, after a cardioversion, his ECG showed pre-excitation suggesting a right-sided posteroseptal AP (Figure 2B). He was on dronedarone 400 mg twice a day and bisoprolol 5 mg twice a day and he was referred for an EP study. No pre-excitation had been recorded during his episodes of atrial fibrillation. In the EP study, the pathway was mapped to be midseptal and it presented only with antegrade conduction, with a basal AP effective refractory period of 300 ms and during isoproterenol provocation 280 ms. No tachycardia could be induced. The pathway was ablated successfully with cryotechnique, and the patient was discharged in normal sinus rhythm with unchanged medication.

### Results

Malignant properties were not demonstrated in either case. Neither was any deterioration of the antegrade AV conduction seen at the time of the EP study. In the first case, the AP was seen soon after aortic valve surgery and in the second case, 3 years after a second aortic operation. In case 1, the AP did not have clinical significance. The AP in the second case was successfully ablated.

## Discussion

We present 2 cases involving patients with previously unknown pre-excitation, in whom pre-excitation was discovered after aortic surgery. Both of the pathways were located on the right side. The incidence of unmasking of an AP after aortic surgery has not been systematically reported.

Deterioration of AV conduction is the likely mechanism facilitating AP conduction and unmasking an AP. The perioperative or postoperative conduction disorders in these cases are most often due to trauma to the AV node, the main bundle, or the left or, alternatively, the right bundle, as the conduction system is situated in the vicinity of the aortic valve. Also, postoperative inflammatory response can account for conduction disturbances. The risk of AV block requiring permanent pacemaker implantation after aortic surgery was 2.5% in a large sample from Mayo Clinic.^7^ Late conduction system abnormalities have been reported in up to 13.7% of patients in an older study.^8^ After transcatheter aortic valve replacement, the risk of AV block leading to pacemaker implantation has been 7%–42.5%,^9^ and a meta-analysis of 41 studies suggested an implantation rate of 17% among 11,210 patients.^10^

In case 1, there were signs of possible perioperative trauma: the ECG at discharge showed nodal rhythm or AV block I with extremely long PQ interval, and the EP study 6 months later showed frequency-dependent left bundle branch block but then otherwise intact AV conduction. In case 2, the AV conduction was found normal 3 years after the surgery. In addition to traumatic or inflammatory causes, negatively dromotropic medication can deteriorate conduction. Both our patients were on beta blockers, and the patient in case 2 also on dronedarone, when the pre-excitation was diagnosed. However, the pre-excitation persisted even when the medication was discontinued.

In addition to deterioration of the AV conduction, the derangement in the sympathovagal balance adjacent to the AP could affect its conductive properties and lead to preferred conduction through the AP instead of the AV node. Destruction of parasympathetic nerve fibers during the surgery or neural remodeling as a result of postoperative sympathetic nerve sprouting might account for such alterations.

It is important to evaluate the significance of a newly discovered pre-excitation after aortic valve intervention. The presence of an AP may protect from a symptomatic AV block necessitating a pacemaker implantation. If full overt pre-excitation masks an advanced AV block, the risk for a total AV block might still exist, because the conduction through an AP can be intermittent or deteriorating with advancing age. The possible malignant electrical properties of an AP cannot be excluded in the case of only intermittent conduction, even if the APs with intermittent pre-excitation show more often benign properties when compared to the overt ones.^11^ Previously unrecognized asymptomatic intermittent APs with malignant properties have been reported in up to 15% of cases studied (ie, revealing a short AP effective refractory period <250 ms or fast pre-excited atrial fibrillation during an EP study).^12^, ^13^, ^14^

## Conclusion

These cases elucidate the importance of recording and interpreting 12-lead ECG after cardiac surgery or other invasive valvular procedures that might affect AV conduction or facilitate conduction in an AP. Our report is the first one to show persistent unmasking of pre-excitation after aortic valve surgery. The patients presenting with pre-excitation should be assessed by noninvasive or invasive methods, in order to characterize the risk of developing malignant arrhythmias or AV block.

**KEY TEACHING POINTS**

• Cardiac surgery may unmask previously unknown pre-excitation, possibly because of deterioration of atrioventricular (AV) conduction. Recording and interpreting a 12-lead electrocardiogram after cardiac surgery and intervention is important to find disturbances in AV conduction or previously concealed accessory pathways.
• Full pre-excitation can mask a high-degree AV block. In such patients, pacemaker therapy has to be considered.
• The conductive properties of an accessory pathway cannot be automatically classified as benign, even if the conduction in the pathway is only intermittent.

## References

[1] Chhabra A., Trikha A., Sharma N. (2003). Unmasking of benign Wolff-Parkinson-White pattern under general anaesthesia. Indian J Anaesth.

[2] Le Manach Y., Charbucinska K.N., Godet G. (2006). Accessory myocardial pathway mimicking an inferior myocardial infarction after major vascular surgery. Eur J Anaesthesiol.

[3] Visman A.G., Hauer R.N., Robles de Medina E.O. (1993). Unmasking of left free wall ventricular preexcitation by His bundle ablation. Br Heart J.

[4] Simmers T.A., Otterspoor L.C., Winter J.B. (2006). Unmasking accessory pathway conduction due to AV block following tricuspid valve replacement. Neth Heart J.

[5] Breaux D.M., Glancy L.D. (2017). Sinus tachycardia with variable QRS morphology. Am J Cardiol.

[6] Priori S.G., Blomstrom-Lundqvist C., Mazzanti A. (2015). 2015 ESC Guidelines for the management of patients with ventricular arrhythmias and the prevention of sudden cardiac death: The Task Force for the Management of Patients with Ventricular Arrhythmias and the Prevention of Sudden Cardiac Death of the European Society of Cardiology (ESC). Endorsed by: Association for European Paediatric and Congenital Cardiology (AEPC). Eur Heart J.

[7] Greason K.L., Lahr B.D., Stulak J.M. (2017). Long-term mortality effect of early pacemaker implantation after surgical aortic valve replacement. Ann Thorac Surg.

[8] Habich J.M., Scherr P., Zerkowski H.R., Hoffmann A. (2000). Late conduction defects following aortic valve replacement. J Heart Valve Dis.

[9] Rivard L., Schram G., Asgar A. (2015). Electrocardiographic and electrophysiological predictors of atrioventricular block after transcatheter aortic valve replacement. Heart Rhythm.

[10] Siontis G.C., Juni P., Pilgrim T. (2014). Predictors of permanent pacemaker implantation in patients with severe aortic stenosis undergoing TAVR: a meta-analysis. J Am Coll Cardiol.

[11] Al-Khatib S.M., Arshad A., Balk E.M. (2016). Risk stratification for arrhythmic events in patients with asymptomatic pre-excitation: a systematic review for the 2015 ACC/AHA/HRS Guideline for the Management of Adult Patients With Supraventricular Tachycardia: A Report of the American College of Cardiology/American Heart Association Task Force on Clinical Practice Guidelines and the Heart Rhythm Society. Circulation.

[12] Obeyesekere M.N., Klein G.J. (2017). Application of the 2015 ACC/AHA/HRS guidelines for risk stratification for sudden death in adult patients with asymptomatic pre-excitation. J Cardiovasc Electrophysiol.

[13] Aleong R.G., Singh S.M., Levinson J.R., Milan D.J. (2009). Catecholamine challenge unmasking high-risk features in the Wolff-Parkinson-White syndrome. Europace.

[14] Robinson K., Rowland E., Krikler D.M. (1988). Latent pre-excitation: exposure of anterograde accessory pathway conduction during atrial fibrillation. Br Heart J.

